# Usefulness of contrast-enhanced multi-detector computed tomography in identifying upper gastrointestinal bleeding: A retrospective study of patients admitted to the emergency department

**DOI:** 10.1371/journal.pone.0266622

**Published:** 2022-04-07

**Authors:** Dongju Kim, Ji Hoon Kim, Dong Ryul Ko, In Kyung Min, Arom Choi, Jin Ho Beom

**Affiliations:** 1 Department of Emergency Medicine, Yonsei University College of Medicine, Seoul, Republic of Korea; 2 Biostatistics Collaboration Unit, Yonsei Biomedical Research Institute, Yonsei University College of Medicine, Seoul, Republic of Korea; Changhua Christian Healthcare System: Changhua Christian Hospital, TAIWAN

## Abstract

Upper gastrointestinal bleeding (UGIB) is a major cause of clinical deterioration worldwide. A large number of patients with UGIB cannot be diagnosed through endoscopy, which is normally the diagnostic method of choice. Therefore, this study aimed to investigate the diagnostic value of multi-detector computed tomography (MDCT) for patients with suspected UGIB. In this retrospective observational study of 386 patients, we compared contrast-enhanced abdominopelvic MDCT to endoscopy to analyze the performance of MDCT in identifying the status, location of origin, and etiology of UGIB. The sensitivity, specificity, positive predictive value (PPV), negative predictive value (NPV), and accuracy were examined. In the assessment of bleeding status, MDCT was able to accurately identify 32.9% (21.9–43.9, 95% confidence interval [CI]) of patients with active bleeding, 27.4% (18.9–35.9, 95% CI) of patients with recent bleeding, and 94.8% (91.8–97.8, 95% CI) of patients without bleeding evidence (P<0.001). MDCT showed an accuracy of 60.9%, 60.6%, and 50.9% in identifying bleeding in the esophagus, stomach, and duodenum, respectively (P = 0.4028). The accuracy in differentiating ulcerative, cancerous, and variceal bleeding was 58.3%, 65.9%, and 56.6%, respectively (P = 0.6193). MDCT has limited use as a supportive screening method to identify the presence of gastrointestinal bleeding.

## Introduction

Acute gastrointestinal bleeding (AGIB) is a major cause of clinical deterioration worldwide, and its mortality rate is reported to range between 21 and 40% in high-risk patients [1]. Based on the location of bleeding in respect with the ligament of Treitz, AGIB is classified as upper or lower, and symptoms vary between these two conditions [2]. Specifically, upper gastrointestinal bleeding (UGIB) occurs in up to 150 per 100,000 patients [3]. The admission of these patients to emergency department (ED) is crucial to rapidly identify the location, gravity, and nature of the bleeding. This information is fundamental to select the appropriate treatment, which varies based on the location and type of the lesion.

Localization of the bleeding site based on symptoms such as melena or hematochezia is not reliable. Therefore, endoscopy is the diagnostic procedure of choice for patients with UGIB, as, in some cases, it allows for the simultaneous and safe localization and hemostatic treatment of the bleeding lesion [4]. However, although the sensitivity and specificity of esophagogastroduodenoscopy (EGD) have been reported to be 92–98% and 30–100%, respectively, Vreeburg et al. [5] reported that up to 24% of patients with UGIB cannot be diagnosed through this method. This is because endoscopic visibility decreases when the bleeding is catastrophic, and the distal duodenum cannot be appropriately assessed.

Therefore, the use of computed tomography (CT) has increased due to its time-efficiency and accuracy for the diagnosis of gastrointestinal bleeding [6–10]. Especially, multi-detector CT (MDCT) has been reported as a promising method in diagnosing AGIB. In fact, in a previous study, MDCT was able to identify the exact site of bleeding in 24/26 patients with clinical signs of AGIB [11].

Currently, MDCT is most often used as a post-endoscopic follow-up diagnostic tool. Only a few prior studies have investigated whether MDCT grants sufficient reliability to be employed as the predominant tool for the diagnosis of UGIB. Therefore, an evaluation of the diagnostic accuracy of this method is necessary. In this study, we examined the diagnostic accuracy of MDCT for the identification of the status, location of origin, and etiology of bleeding in patients who underwent MDCT prior to endoscopy.

## Materials and methods

### Ethics statement

The study was conducted according to the tenets of the Declaration of Helsinki as revised in 2013 and approved by the institutional review board of Yonsei University Health System Clinical Trial Center (approval number, 4–2021–1093). The institutional review board of Yonsei University Health System Clinical Trial Center waived the requirement for informed consent owing to the retrospective observational design of the study.

### Study design and patient selection

This retrospective observational study analyzed the electronic medical records of patients with suspected UGIB who were admitted to the ED of a single-tertiary teaching hospital from January 2018 to December 2019. Patients over 18 years of age were enrolled if they had complaints of hematemesis, melena, and/or hematochezia, and had undergone MDCT. Patients were excluded if they had undergone endoscopy 24 h after ED admission, or their endoscopic examination had failed. In addition, patients with lower gastrointestinal lesions in the small intestines or colorectum as reported after colonoscopy or sigmoidoscopy were excluded. After MDCT, emergency endoscopy was performed to detect and treat the bleeding regardless of MDCT results.

### Data collection

The gastroenterologists or emergency physicians on duty at the time of patient admission decided whether to perform MDCT as the initial diagnostic test for UGIB. Senior radiologists with over 10 years’ experience and junior radiologists with 2–3 years’ experience analyzed the MDCT images within 3 h after examination without any knowledge of endoscopic results. The presence of active contrast extravasation was defined as active bleeding, while hemorrhagic content, suspicious hematoma, and blood clots were categorized as recent bleeding. MDCT-negative patients were defined as patients without evidence of active or recent bleeding. On endoscopy (EGD, colonoscopy, and sigmoidoscopy), active bleeding was defined as current, oozing, or spurting bleeding, and recent bleeding was defined as stigmata or blood clots without active bleeding. Acute bleeding was defined as the combination of active bleeding and recent bleeding. Data were collected as true positive (TP), true negative (TN), false positive (FP), and false negative (FN).

### MDCT protocol

Patients were scanned with a 64-channel GE Revolution Evo ES CT Scanner (GE healthcare, Milwaukee, WI, USA). Axial images were acquired with maximum intensity projection, and multiplanar reconstructions were performed for 3-mm thick sections for diagnostic interpretation. Scans covered the whole abdomen, from the diaphragm to the lesser trochanter, and were performed with and without contrast. For contrast-enhanced MDCT, arterial- and/or portal-venous phase images were acquired after intravenous injection of Omnipaque 250 contrast medium (Daiichi-Sankyo, Tokyo, Japan) and Radisense 300 (Hoffman Health Pakistan Ltd, Lahore, Pakistan). Patients received 2.0 mL/kg of iodinated contrast media with 300 mg/mL iodine through an automated injector at a rate of 1.0 mL/s. Patients with acute or chronic kidney injury, or iodinated-contrast allergy were excluded from undergoing enhanced MDCT to avoid potential side effects. The MDCT protocol took less than 5 minutes in average.

### Data analysis

The symptomatic patients with gastrointestinal bleeding who initially underwent MDCT followed by endoscopy within 24 hours after admission were included in the final analyses. Only upper gastrointestinal lesions reported after EGD were included. The upper gastrointestinal tract was divided into 3 parts: the esophagus, stomach, and duodenum. In addition, endoscopic diagnoses were divided into three groups: ulcerative, variceal, and cancerous bleeding. Patients with diagnoses other than these three categories were excluded from the analysis. According to their bleeding status, patients with suspected UGIB were categorized into an active, recent, or no bleeding group.

The main purpose of performing MDCT in UGIB is to determine if there is active bleeding that requires emergency intervention. Therefore, data of entire cohort were used to analyze the diagnostic performance. However, when diagnosing the etiology, it is important to identify promptly the lesion that is most common and has higher mortality. Therefore, subgroup analysis was performed based on three diagnoses, which are the most common diagnosis of UGIB: peptic ulcer disease, the second most common diagnosis; esophagogastric varices; and neoplasm, which is increasing recently [12, 13].

The accuracy of MDCT was analyzed based on the agreement of the diagnoses performed through MDCT and EGD. To do this, endoscopy was established as the gold standard. Specifically, the diagnostic performance of MDCT was measured by calculating the sensitivity, specificity, positive predictive value (PPV), negative predictive value (NPV), and accuracy. Continuous variables were given as median with interquartile range (IQR), and the categorical variables as proportions. Sensitivity, specificity, PPV, NPV, and accuracy were calculated with a 95% confidence interval (CI). True positive (TP) is defined as the number of cases correctly identified as patients with disease; false positive (FP) as the number of cases incorrectly identified as patient with disease; true negative (TN) as the number of cases correctly identified as non-diseased; and false negative (FN) as the number of cases incorrectly identified as non-diseased. The accuracy is its power to discriminate the patients with disease and without disease accurately. To calculate the accuracy, the proportion of true positive and true negative in all cases were calculated as (TP+TN)/(TP+TN+FP+FN). The sensitivity, which was defined as the ability to differentiate the patients with disease correctly, was calculated as the proportion of true positive cases in patients with disease and was calculated as TP/(TP+FN). The specificity means the ability to differentiate patients without disease correctly and was calculated as TN/(TN+FP). P values < 0.05 were considered to be statistically significant. The R package (version 3.6.0, The R Foundation for Statistical Computing, Vienna, Austria) and SAS software (version 9.4, SAS Inc., Cary, NC, USA) were used for analysis.

## Results

### Patient characteristics

**Fig 1:**
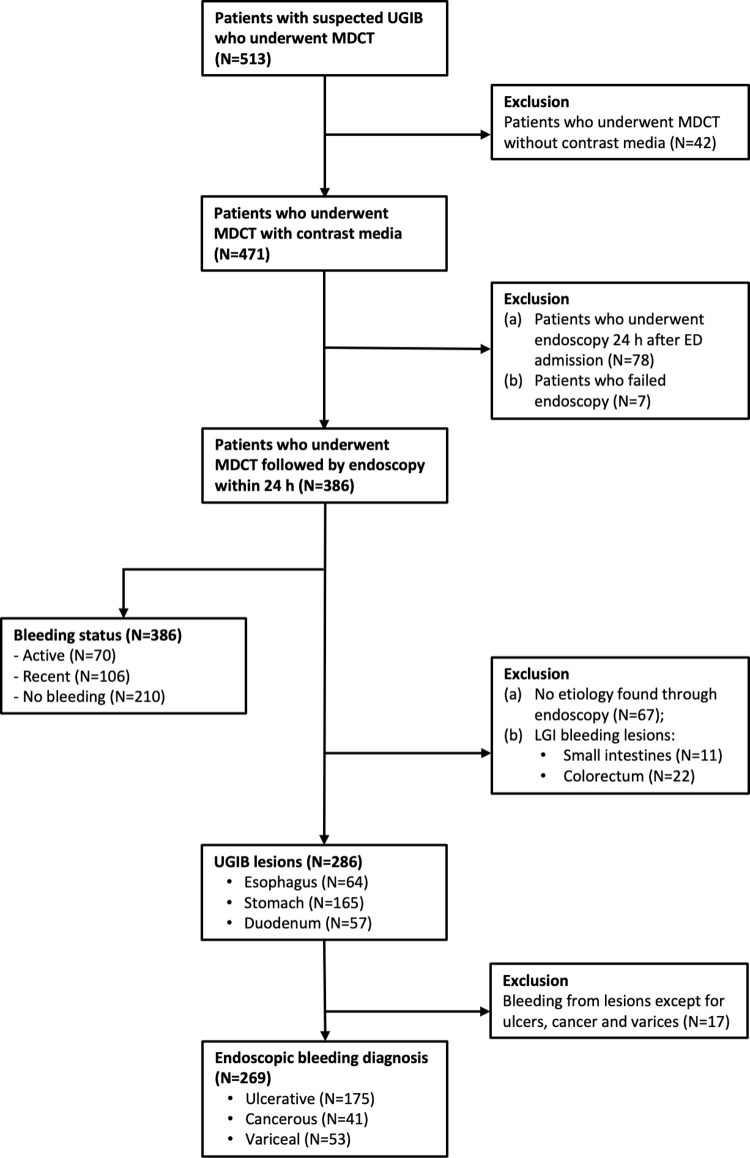
Study flow chart. UGIB, upper gastrointestinal bleeding; MDCT, multi-detector computed tomography; ED, emergency department; LGI, lower gastrointestinal bleeding.

A total of 471 patients with suspected gastrointestinal bleeding initially underwent contrast-enhanced MDCT, but 78 patients who underwent endoscopy after 24 hours after admission and 7 patients who failed endoscopy were excluded. A total of 386 patients were enrolled. Among these, 70 patients presented with active bleeding, 106 with recent bleeding, and 210 did not have any evidence of bleeding after endoscopy. In addition, 67 patients who showed no definite etiology of bleeding, 33 with bleeding in the small intestines or colorectal area, and 17 with lesions other than ulcers, cancer and varices were excluded. Ultimately, 269 patients were included in the final analyses. Fig 1 summarizes the study flow.

### The diagnostic performance of MDCT in identifying current bleeding status

Among the 386 patients who underwent EDG and MDCT within 24 h after admission, 70 patients were diagnosed with active bleeding through endoscopy, but only 23 were accurately identified through MDCT. In addition, out of the 106 patients confirmed to have experienced recent bleeding, MDCT was able to solely recognize 29 patients. According to MDCT results, 11 patients were suspected to have active bleeding, but no bleeding was confirmed through endoscopy. The accuracy of MDCT to diagnose bleeding status was 32.9% (95% CI: 21.9–43.9) in patients with active bleeding, 27.4% (195% CI: 8.9–35.9) in those with recent bleeding, and 94.8% (95% CI: 91.8–97.8,) in individuals without bleeding (P = 0.001). No statistical difference in accuracy was detected in the differentiation between active and recent bleeding (P>0.99) (Table 1).

**Table 1:** The diagnostic performance of MDCT in identifying bleeding status.

	Bleeding status				P value			
	Active	Recent	None	Total	Overall	1 vs. 2	1 vs. 3	2 vs. 3
	(N = 70)	(N = 106)	(N = 210)					
**TP**	23	29	0	52				
**TN**	0	0	199	199				
**FP**	0	0	11	11				
**FN**	47	77	0	124				
**Sensitivity**	32.9	27.4	-	29.6	-	0.4339	-	-
**(95% CI)**	(21.9, 43.9)	(18.9, 35.9)		(22.8, 36.3)				
**Specificity**	-	-	94.8	94.8	-	-	-	-
**(95% CI)**			(91.8, 97.8)	(91.8, 97.8)				
**PPV**	100.0	100.0	0.0	82.5	<0.0001	>0.9999	<0.0001	<0.0001
**(95% CI)**	(100.0, 100.0)	(100.0, 100.0)	(0.0, 0.0)	(73.2, 91.9)				
**NPV**	0.0	0.0	100.0	61.6	<0.0001	>0.9999	<0.0001	<0.0001
**(95% CI)**	(0.0, 0.0)	(0.0, 0.0)	(100.0, 100.0)	(56.3, 66.9)				
**Accuracy**	32.9	27.4	94.8	65.0	<0.0001	0.4339	<0.0001	<0.0001
**(95% CI)**	(21.9, 43.9)	(18.9, 35.9)	(91.8, 97.8)	(60.3, 69.8)				

TP, true positive; TN, true negative; FP, false positive; FN, false negative; CI, confidence interval; PPV, positive predictive value; NPV, negative predictive value; 1, active bleeding; 2: Recent bleeding; 3: No bleeding

### The diagnostic performance of MDCT in identifying the location of bleeding lesions

Among the 286 patients with UGI bleeding lesions, abdominopelvic MDCT was able to successfully identify 9 out of 64 patients with bleeding in the esophagus, 34 out of 165 with bleeding in the stomach, and 10 out of 57 with lesions in the duodenum. On the other hand, endoscopy was able to differentiate 32, 87, and 38 patients with bleeding in the esophagus, stomach, and duodenum, respectively. The sensitivity and specificity of MDCT to correctly assign the bleeding site to the esophagus were 25.0% (95% CI: 10.0–40.0) and 96.9% (95% CI: 90.9–102.9) respectively. The sensitivity and specificity to identify bleeding in the stomach were 32.2% (95% CI: 22.4–42.0,) and 92.3% (95% CI: 86.4–98.2), respectively. For bleeding in the duodenum, the sensitivity and specificity of MDCT were 26.3% (95% CI: 12.3–40.3) and 100%, respectively. The accuracy of MDCT in correctly identifying the location of bleeding lesions was 60.9%, 60.6%, and 50.9% for the esophagus, stomach, and duodenum, respectively (P = 0.4028) (Table 2).

**Table 2:** The diagnostic performance of MDCT in identifying the location of bleeding lesions.

	Bleeding location			P value			
	Esophagus	Stomach	Duodenum	Overall	1 vs. 2	1 vs. 3	2 vs. 3
	(N = 64)	(N = 165)	(N = 57)				
**TP**	8	28	10				
**TN**	31	72	19				
**FP**	1	6	0				
**FN**	24	59	28				
**Sensitivity**	25.0	32.2	26.3	0.6709	0.4494	0.9001	0.5118
**(95% CI)**	(10.0, 40.0)	(22.4, 42.0)	(12.3, 40.3)				
**Specificity**	96.9	92.3	100.0	0.3327	0.3728	0.4364	0.212
**(95% CI)**	(90.9, 102.9)	(86.4, 98.2)	(100.0, 100.0)				
**PPV**	88.9	82.4	100.0	0.3429	0.6367	0.2788	0.1529
**(95% CI)**	(68.4, 109.4)	(69.5, 95.2)	(100.0, 100.0)				
**NPV**	56.4	55.0	40.4	0.1834	0.8607	0.1085	0.0872
**(95% CI)**	(43.3, 69.5)	(46.4, 63.5)	(26.4, 54.5)				
**Accuracy**	60.9	60.6	50.9	0.4028	0.9632	0.2656	0.1993
**(95% CI)**	(49.0, 72.9)	(53.2, 68.1)	(37.9, 63.9)				

TP, true positive; TN, true negative; FP, false positive; FN, false negative; CI, confidence interval; PPV, positive predictive value; NPV, negative predictive value; 1, esophagus; 2, stomach; 3, duodenum

### The diagnostic performance of MDCT in identifying the etiology of bleeding

Among the final 269 patients, a total of 175, 41, and 53 patients were diagnosed with ulcerative, cancerous, and variceal bleeding by endoscopic evaluation, respectively. Of these, MDCT was able to correctly diagnose 36, 4, and 7 patients, while endoscopy successfully identified 101, 18, and 26, respectively. The sensitivity and specificity of MDCT in identifying ulcerative bleeding were 31.7% (95% CI: 22.6–40.8,) and 94.6% (89.4–99.8, 95% CI), respectively. The specificity and sensitivity to recognize cancerous bleeding were 22.2% (95% CI: 3.0–41.4) and 100%, respectively, while for variceal bleeding, they corresponded to 19.2% (95% CI: 4.1–34.4) and 92.6% (95% CI: 82.7–102.3), respectively. The accuracy of MDCT in diagnosing ulcerative, cancerous, and variceal bleeding was 58.3%, 65.9%, and 56.6%, respectively (P = 0.6193) (Table 3).

**Table 3:** The diagnostic performance of MDCT in identifying the etiology of bleeding.

	Endoscopic diagnosis			P value			
	Ulcerative	Cancerous	Variceal	Overall	1 vs. 2	1 vs. 3	2 vs. 3
	(N = 175)	(N = 41)	(= 53)				
**TP**	32	4	5				
**TN**	70	23	25				
**FP**	4	0	2				
**FN**	69	14	21				
**Sensitivity**	31.7	22.2	19.2	0.3768	0.4208	0.2127	0.8089
**(95% CI)**	(22.6, 40.8)	(3.0, 41.4)	(4.1, 34.4)				
**Specificity**	94.6	100.0	92.6	0.4475	0.2548	0.7064	0.1828
**(95% CI)**	(89.4, 99.8)	(100.0, 100.0)	(82.7, 102.5)				
**PPV**	88.9	100.0	71.4	0.3256	0.4822	0.2225	0.2373
**(95% CI)**	(78.6, 99.2)	(100.0, 100.0)	(38.0, 104.9)				
**NPV**	50.4	62.2	54.4	0.4343	0.2012	0.639	0.4736
**(95% CI)**	(42.1, 58.7)	(46.5, 77.8)	(40.0, 68.7)				
**Accuracy**	58.3	65.9	56.6	0.6193	0.3738	0.828	0.3627
**(95% CI)**	(51.0, 65.6)	(51.3, 80.4)	(43.3, 70.0)				

TP, true positive; TN, true negative; FP, false positive; FN, false negative; CI, Confidence interval; PPV, positive predictive value; NPV, negative predictive value; 1, ulcerative; 2, cancerous; 3, variceal

## Discussion

In this study, we have identified that MDCT has significantly low sensitivity for the detection of UGIB, which was finally diagnosed by endoscopic evaluation. Therefore, MDCT is not recommended as a sole method for diagnosing UGIB, or either as the dominant diagnostic method for medical decision making. Overall, MDCT cannot replace endoscopy. Nonetheless, EGD has considerable limitations in the diagnosis of patients with massive bleeding or other co-morbidities, and often fails to assess the source of bleeding. In this aspect, MDCT was considered to be an outstanding diagnostic method due to its rapidity, spectrum of availability, and minimal invasiveness [14–16]. A prospective study of patients with UGIB who underwent EGD after MDCT showed that the sensitivity of this latter methodology to identify the location and the etiology of bleeding was 100% and 90.9%, respectively [17]. Moreover, Jaeckle et al reported the accuracy of MDCT for detection and localization of acute upper and lower GI hemorrhage [11]. In this study, overall sensitivity was 88% and specificity was 100%, which were higher than those in our study. However, we included all patients with symptomatic gastrointestinal bleeding who underwent EGD at any time within 24hours after performing MDCT according to our study protocol. GI bleeding, which was shown on MDCT may have stopped at the time of endoscopy: in these instances, the degree and type of bleeding will be differently detected and diagnosed. In fact, Miller et al has reported six cases of bleeding identified by MDCT but not through other modalities of diagnosis [18]. The sensitivity might be overestimated since the bleeding rate may vary depending on the lesion and location and the bleeding is not continuous in nature [19, 20].

Several studies have dealt with the use of CT in lower GI bleeding as an alternative diagnostic tool since. several different diagnostic modalities such as angiography, 99mTc- RBC scintigraphy, or colonoscopy can be used as the standard procedure for lower GI bleeding, [21–23]. However, for UGIB, endoscopy is the procedure which is considered as the first diagnostic method and treatment of choice. Therefore, it is important to rapidly detect patients who need prompt treatment with limited resources in the ED, MDCT can be performed first to identify the urgency and determine treatment modality of patients. In this study, unstable patients who were unable to move for CT or endoscopy or needed application of Senstaken–Blakemore tube first before 24 hours of endoscopy for hemostasis of massive esophageal variceal bleeding and stable patients without current bleeding could not be included due to the absence of endoscopic evaluation. In other words, all patients for whom emergency endoscopy was considered by the physician were included. Since previous studies included patients suspected of obscure bleeding rather than symptomatic current bleeding, it is inappropriate to apply the result suggested in the previous study to the environment of ED [18, 24]. Moreover, contrary to what has been previously reported, the results of our study indicated that MDCT has relatively low diagnostic sensitivity to identify the site and etiology of bleeding; the reason for this discrepancy could be due to the fact that, in all the cases included in our study, the extent of bleeding was rather limited, and, as such, it was unlikely to be detected through MDCT.

**Fig 2:**
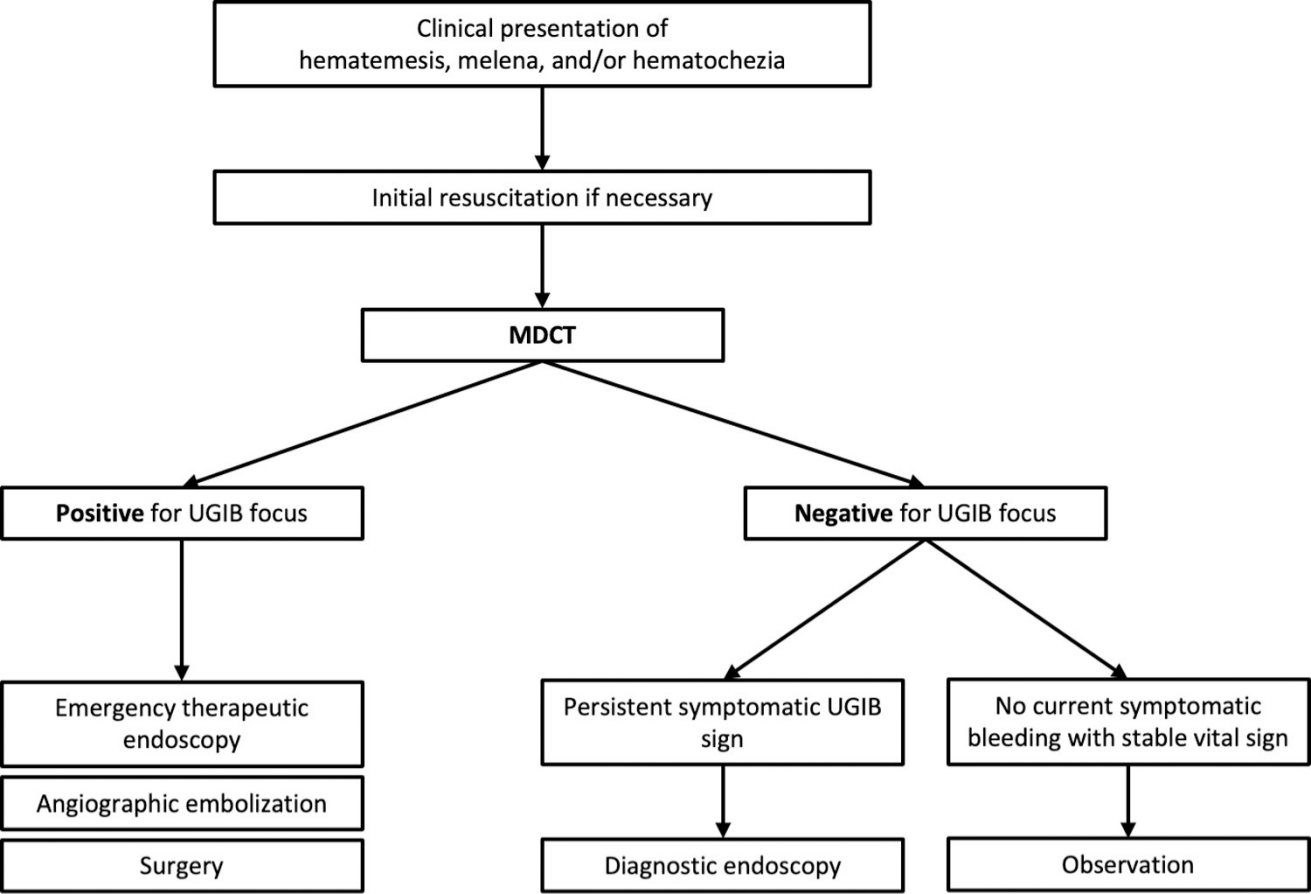
The flow diagram to use MDCT in UGIB. UGIB, upper gastrointestinal bleeding; MDCT, multi-detector computed tomography.

Regardless, the conclusions of this study will have a fundamental role in clinical practice. Firstly, there are ambiguous situations in which the patient’s symptoms cannot be exclusively employed to assess bleeding status. In addition, in several cases, laboratory tests and the patient’s vital signs have also limited accuracy in estimating the nature of bleeding. Because MDCT showed high specificity in this study, this technique could be used as a rapid and non-invasive tool to supplement clinical symptom assessments and laboratory tests. This could be a feasible course of action especially in some countries such as South Korea, where MDCT is inexpensive and can be easily performed in the ED of any hospital. In this study, when the radiologist reported if the patients bled in upper gastrointestinal tract in the APCT, the positive predictive value was 100%, which can be interpreted that if bleeding was seen on CT, bleeding was actually present in all cases in EGD as well. Therefore, the use of CT can be summarized through the flow diagram (Fig 2).

Therefore, we recommend performing MDCT for rapid screening before endoscopy in patients with suspected UGIB. Moreover, in the cases in which MDCT successfully identifies the location or etiology of bleeding, we suggest to immediately commence hemostatic treatments, such as embolization and endoscopic hemostasis [25].

However, it is fundamental that physicians be mindful of the several limitations of MDCT. Firstly, this methodology can solely visualize the location and status of the bleeding but cannot act as a direct therapeutic option like endoscopy can. Moreover, if the patient is allergic to contrast media or has underlying renal failure, contrast-enhanced MDCT cannot be employed [26].

This study has a few limitations. First, due to the retrospective nature of the research design, unidentified confounders and selection bias might have been introduced. In addition, not only EGD could not be performed on critically ill patients, but also MDCT is not a viable option in individuals with allergies to contrast media, or with acute or chronic kidney injury. Moreover, the non-blinded data interpretation carried out by different radiologists might have generated variation in the reported findings. Furthermore, since this research was conducted at a single-tertiary hospital, multi-center studies are needed. Finally, FP and FN results could have been produced due to the widely diverse characteristic of UGIB.

## Conclusions

In this study, we analyzed the diagnostic ability of contrast enhanced MDCT to identify the status, location, and etiology of bleeding in patients with suspected UGIB. Early identification of these factors is essential for these individuals, so that hemostatic treatments and patient stabilization can be promptly initiated [27]. We suggest that, if MDCT is a cost-effective option, this examination can be easily performed, but should only be intended as a supplemental tool to determine the presence of bleeding.

## Supporting information

S1 File Anonymized data set for the study. (XLSX)
